# Consumption of dietary fiber and *APOA5* genetic variants in metabolic syndrome: baseline data from the Korean Medicine Daejeon Citizen Cohort Study

**DOI:** 10.1186/s12986-024-00793-0

**Published:** 2024-04-05

**Authors:** Jimi Kim, Younghwa Baek, Siwoo Lee

**Affiliations:** 1 Department of Food and Nutrition, Changwon National University, 20 Changwondaehak-ro, Uichang-gu, 51140 Changwon, Gyeongnam South Korea; 2 Korean Medicine Data Division, Korea Institute of Oriental Medicine, 1672 Yuseong-daero, Yuseong-gu, 34054 Daejeon, South Korea

**Keywords:** Metabolic syndrome, fiber, *APOA5* gene, Gene-diet interaction

## Abstract

**Background:**

Consumption of dietary fiber has been suggested as an important aspect of a healthy diet to reduce the risk of metabolic syndrome (MetS), including cardiovascular disease. The role of fiber intake in MetS might differ by individual genetic susceptibility. *APOA5* encodes a regulator of plasma triglyceride levels, which impacts the related mechanisms of MetS. This study investigated the association between dietary fiber and the risk of MetS, assessing their associations according to *APOA5* genetic variants.

**Methods:**

A total of 1985 participants aged 30–55 years were included from a cross-sectional study based on the Korean Medicine Daejeon Citizen Cohort study at baseline (2017–2019). Dietary fiber intake was measured using a semiquantitative food frequency questionnaire. The *APOA5* polymorphisms (rs2266788 A > G, rs662799 A > G, and rs651821 T > C) were genotyped using the Asia Precision Medicine Research Array. Logistic regression was used to estimate odds ratios (ORs) and 95% confidence intervals (95% CIs).

**Results:**

A higher consumption of dietary fiber was associated with a lower prevalence of MetS (*P* = 0.025). Among the components of MetS, an inverse association with dietary fiber was observed in increased waist circumference (OR, 95% CI = 0.60, 0.41–0.88, *P* for trend = 0.009) and elevated triglycerides (OR, 95% CI = 0.69, 0.50–0.96, *P* for trend = 0.012). Regarding the interaction with *APOA5* genetic variants, a stronger association with dietary fiber intake was shown in G allele carriers of rs662799 than in A/A carriers (OR, 95% CI = 2.34, 1.59–3.44, *P* for interaction = 0.024) and in C allele carriers of rs651821 than in T/T carriers (OR, 95% CI = 2.35, 1.59–3.46, *P* for interaction = 0.027).

**Conclusions:**

The findings of this study suggest that the benefits of dietary fiber on the risk of MetS could be modified by genetic variants of the *APOA5* gene, providing a more effective strategy for preventing MetS.

**Supplementary Information:**

The online version contains supplementary material available at 10.1186/s12986-024-00793-0.

## Introduction

Dietary fiber has long been known to contribute to improving and maintaining a healthy life since noncommunicable diseases have been rapidly increasing [1]. According to the Codex Alimentarius, dietary fiber was defined as nondigestible carbohydrate polymers with ten or more monomeric units in the human digestive tract [1, 2]. Natural fiber is obtained from plant-based foods such as fruits, vegetables, legumes, and whole grains. Fiber is subclassified into insoluble (e.g., cellulose, hemicellulose, and lignin) and soluble fiber (e.g., pectin, β-glucan, gums, and mucilage), resistant starch, and prebiotics based on solubility. To maintain and deliver health benefits such as the reduced risk of obesity, diabetes, cardiovascular disease, and some types of cancer, an adequate intake of dietary fiber was suggested, and the daily recommended amounts of fiber intake differ by sex and age [1, 3]. The range of global recommended dietary fiber is from 25 to 38 g per day, which could be achieved through the consumption of a variety of plant-based foods, fruits, and vegetables [1]. In noncommunicable diseases, accumulating evidence has accounted for the role of dietary fiber in assessing the optimal diet strategy for metabolic disorders and its underlying mechanism, which includes modulating body weight, lipid reduction, glucose metabolism, blood pressure, and chronic inflammation [4].

Metabolic syndrome (MetS) refers to the concurrence of five components relevant to cardiovascular risk factors, including elevated waist circumference (WC), triglycerides (TG), peripheral blood pressure (BP), fasting blood glucose (FBG), and low high-density lipoprotein cholesterol (HDL-C), resulting in abdominal obesity, dyslipidemia, hypertension, and hyperglycemia [4, 5]. MetS increases the risk of cardiovascular disorders, type 2 diabetes mellitus, stroke, and several cancers [6, 7]. Habitual diet and lifestyle are important contributing factors to the development of MetS [8]. Researchers in numerous epidemiological studies have reported that a healthy high fiber diet is necessary to prevent the risk of MetS. In a recent systematic meta-analysis, the consumption of fiber was found to be associated with a lower likelihood of MetS, indicating a possible inverse association between dietary fiber intake and the risk of MetS [3, 9]. To understand the effect of fiber and prevent MetS, it is necessary to identify the interaction with genetic susceptibility that impacts the related mechanisms of MetS [10]. Nevertheless, there are limited studies that have examined the interactive effect between dietary fiber intake and specific genes based on metabolism related to MetS.

Among the genetic mutations affecting MetS, apolipoprotein A5 (*APOA5*) is one of the genes in the *APOA1/C3/A4/A5* gene cluster at 11q23. The *APOA5* gene is a component of lipoprotein fractions that encode a protein-coding gene linked to determining plasma triglyceride levels [11]. The *APOA5* gene may have functions that modulate the activation of lipoprotein lipase, secretion of very low-density lipoprotein (VLDL) particles, and interaction of the low-density lipoprotein (LDL) receptor family, which are implicated in MetS and thereby lead to an increased risk of coronary artery disease [12–14]. Recently, genome-wide association studies (GWAS) reported common genetic variants of *APOA5* for MetS susceptibility according to the GWAS catalog (www.ebi.ac.uk/gwas/) [15–19]. Among the single nucleotide polymorphisms (SNPs) in *APOA5*, the − 1131T > C variant (rs662799) in the promoter region was specifically associated with hyperglyceridemia and resulted in an increased risk of MetS, depending on the different populations [18]. In addition, a recent meta-analysis revealed the impact of the rs662799 variant on MetS [20]. A few studies have demonstrated the interactive effects of modifiable lifestyle factors, including smoking, alcohol consumption, and habitual diet, on nutrients in food (e.g., carbohydrates, fats, n-6 fatty acids, whole grains, legumes, and intake of red and processed meat) according to different genotypes of the rs662799 variant in the *APOA5* gene [21–25]. Given that a variety of environmental risk factors contribute to the occurrence of MetS, however, evidence of associations between the common variant of the *APOA5* gene and risk factors for MetS, particularly dietary fiber, is still insufficient.

Taken together, this study considers the common SNPs (rs2266788, rs662799, and rs651821) in the *APOA5* gene for MetS susceptibility identified by previous GWAS [15–19]. The aim of the study was to examine the association between dietary fiber intake and MetS along with its components and to explore how the role of dietary fiber in MetS could be modified by genetic variants of the *APOA5* gene.

## Materials and methods

### Study population

**Fig. 1:**
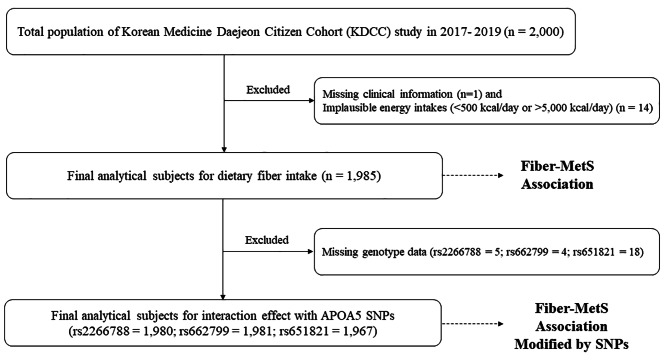
Flow diagram of analytical sample: the KDCC study

This cross-sectional study was based on the Korean Medicine Daejeon Citizen Cohort (KDCC) study, which is a prospective cohort study in Korea aimed at assessing the causal relationships between lifestyle, genomics, and chronic diseases according to Korean medicine types. The KDCC study began in 2017 and is an ongoing prospective study of 2,000 adults aged 30–55 years living in Daejeon. The baseline survey was conducted from 2017 to 2019, with three follow-ups every 2 years between 2020 and 2025. The details of the KDCC study regarding the rationale, design, and baseline characteristics are described elsewhere [26]. Of the 2,000 subjects who completed a structured questionnaire and physical examination, one participant was excluded due to missing clinical information, and 14 participants were excluded because of implausible energy intake (< 500 kcal/day or > 5,000 kcal/day). Therefore, a total of 1,985 participants were included to analyze the association between dietary fiber intake and the risk of MetS. Additionally, individuals for whom a blood sample was not available to examine the individual’s DNA sequence were excluded from the association between genetic variants and their interactions with dietary fiber intake regarding MetS. For the genetic association, 1,980 participants for rs2266788, 1,981 participants for rs662799, and 1,967 participants for rs651821 were selected (Fig. 1). All participants provided written informed consent prior to participation, and the study protocol was approved by the Institutional Review Board at the Korea Institute of Oriental Medicine (IRB No. I-1703/002–002) and Dunsan Korean Medicine Hospital of Daejeon University (IRB No. DJDSKH-17-BM-12).

### Assessment of dietary fiber intake and covariates

All participants completed structured questionnaires regarding their sociodemographic factors, lifestyle, and dietary habits, which were administered by a trained interviewer. The participant’s habitual dietary intake was assessed using a semiquantitative food frequency questionnaire (SQFFQ) consisting of the usual frequencies of consumption classified into nine categories ranging from never or rare to three times per day and portion sizes consumed limited to three or four specified serving sizes [27]. Daily energy and fiber intake were calculated using CAN-PRO 5.0 (Computer Aided Nutritional Analysis Program, The Korean Nutrition Society, Seoul, Korea). The sociodemographic factors included age (continuous), sex, BMI (< 25 kg/m^2^ or ≥ 25 kg/m^2^), education level, occupation, and income. Health-related lifestyle factors included alcohol consumption, smoking status, and physical activity. Alcohol consumption was classified as none, ex-drinker, and current drinker. Smoking status was categorized as none, ex-smoker, and current smoker. Physical activity was assessed using the Korean version of the Global Physical Activity Questionnaire, and total units of metabolic equivalent task (MET-min/week) were calculated [28, 29]. Based on MET values, physical activity was subclassified into three groups (low, moderate, or high).

### Diagnosis of metabolic syndrome

The National Cholesterol Education Program-Adult Treatment Panel III (NCEP-ATP III) [30] with Korean-specific cutoffs of WC based on the guidelines of the Korean Obesity Society [31] was used to assess the diagnosis of MetS. Participants who met three or more of the following clinical criteria of 5 components were diagnosed with MetS: (1) abdominal obesity based on WC with cutoff points specific to South Koreans (≥ 90 cm in men and ≥ 85 cm in women); (2) TG levels (≥ 150 mg/dL or specific treatment for lipid abnormality); (3) BP (systolic BP, ≥ 130 mmHg and diastolic BP, ≥ 85 mmHg), or treatment for diagnosed hypertension; (4) FPG level (≥ 100 mg/dL or diagnosed type 2 diabetes); and (5) HDL-C level (< 40 mg/dL in men and < 50 mg/dL in women or drug treatment for lipid abnormality).

### Genotyping

The *APOA5* polymorphisms (rs2266788 A > G, rs662799 A > G, and rs651821 T > C) were selected from the genome-wide significant SNPs associated with MetS (Supplementary Table S1). Genomic DNA was extracted, and genotyping was performed using the Axiom™ Asia Precision Medicine Research Array (PMRA) chip (Thermo Fisher Scientific, Waltham, MA, USA), which includes over 750,000 SNPs containing 50,000 novel markers that can cover East and South Asian populations based on the human reference genome (GRCh37). Genotyping was successfully performed for 1,980 subjects for rs2266788, 1,981 subjects for rs662799, and 1,967 subjects for rs651821 (Fig. 1).

### Statistical analyses

To assess dietary fiber consumption, energy-adjusted fiber intake was estimated using residual methods from a regression model with total caloric intake [32]. The energy-adjusted dietary fiber intake was divided into quartile groups to investigate the associations with the risk of MetS. Differences in sociodemographic and lifestyle factors, as well as MetS risk with its components, were described as the means and SD for continuous variables and frequencies and percentages for categorical variables across the quartile groups of dietary fiber intake. Linear regression was used to determine trends for BMI; the Jonckheere-Terpstra test was used for other continuous variables, and the Cochran-Mantel-Haenszel test was used for categorical variables as appropriate. Odds ratios (ORs) and 95% confidence intervals (95% CIs) were estimated from multivariable logistic regression methods. The multivariable models were adjusted for a continuous variable (age) and categorical variables (sex, BMI, education level, occupation, income, alcohol consumption, smoking status, and physical activity) as described previously. The median intake of each quartile of fiber intake was used as a continuous variable to test for linear trends. In the analysis of *APOA5* genetic variants, the χ^2^ test was used to assess Hardy-Weinberg equilibrium (HWE) of *APOA5* polymorphisms (rs2266788, rs662799, and rs651821) in non-MetS groups. To determine gene-diet interactions, the dietary intake of fiber was divided into two groups (high and low) based on the median intake levels in the non-MetS groups. The likelihood ratio test comparing the main effects of the model including interaction terms was used to examine gene-diet interactions. For power analyses, sufficient power calculations were performed, assuming a gene-diet interaction effect of *APOA5* genetic variants (OR = 2.3) based on the assumptions of genetic effects (OR = 2.2) and environmental effects (OR = 0.8) using Quanto version 1.2.4 with 80% at an α level of 0.05. SAS version 9.4 (SAS Institute Inc., Cary, NC, USA) was used to perform all statistical analyses. Statistical significance was defined as a two-sided *p*-value less than 0.05.

## Results

### General characteristics of the study population according to quartile group of dietary fiber intake

Table 1 shows the general characteristics of the study population, including sociodemographic factors, lifestyle, daily energy intake, MetS and its components, categorized into quartile groups of dietary fiber intake. Participants with higher fiber intake were relatively older than those with the lowest fiber intake (*P* < 0.001). The consumption of dietary fiber varied by sex, with higher fiber intake observed in females than in males (*P* < 0.001). Participants in the highest quartile had a lower BMI than those in the lowest quartile (*P* = 0.008). Across quartiles, participants with higher fiber intake were less likely to be current alcohol consumers, current smokers, or highly exercised (*P* < 0.001). However, daily total energy intake showed no significant association. Participants with higher fiber intake were less likely to have MetS risk (*P* = 0.045). Except for the FBG level, the mean WC (*P* < 0.001), TG (*P* < 0.001), systolic BP (*P* = 0.004), and diastolic BP (*P* < 0.001) were significantly higher in the lowest quartile of fiber consumers than in those with higher fiber intake; however, the HDL-C level was increased (*P* = 0.002).

**Table 1:** Baseline characteristics of the study population according to quartile group of dietary fiber intake, the KDCC study, 2017–2019

	Q1	Q2	Q3	Q4	*P* for trend
Dietary fiber (g/day)	(< 17.89)	17.89-<20.10	20.10-<22.74	≥ 22.74	
*N*	497	495	496	497	
Age (years)	42.7 ± 6.9	43.8 ± 6.7	44.0 ± 6.8	45.0 ± 6.7	< 0.001
Sex (*n*, %)					
Male	228 (45.9)	149 (30.1)	152 (30.7)	77 (15.5)	< 0.001
Female	269 (54.1)	346 (69.9)	344 (69.4)	420 (84.5)	
BMI (kg/m^2^) (*n*, %)	24.66 ± 3.62	24.45 ± 3.66	24.32 ± 3.43	23.99 ± 3.55	0.008
< 25	295 (59.4)	299 (60.4)	295 (59.5)	326 (65.6)	0.14
≥ 25	202 (40.6)	196 (39.6)	201 (40.5)	171 (34.4)	
Education level (*n*, %)					
Middle school or less	4 (0.8)	4 (0.8)	5 (1.0)	6 (1.2)	0.17
High school	155 (31.2)	164 (33.1)	173 (34.9)	197 (39.6)	
College or more	335 (67.4)	323 (65.3)	314 (63.3)	294 (59.2)	
Occupation (*n*, %)					
Professionals, administrative, management, or office jobs	208 (41.9)	160 (32.3)	150 (30.2)	151 (30.4)	< 0.001
Sales or service positions	131 (26.4)	139 (28.1)	155 (31.3)	143 (28.8)	
Agriculture, manufacturing, mining, or army service	47 (9.5)	24 (4.9)	42 (8.5)	30 (6.0)	
Housekeeping, unemployment, or others	111 (22.3)	172 (34.8)	149 (30.0)	173 (34.8)	
Income (10,000 won/month) (*n*, %)					
<200	39 (7.9)	29 (5.9)	30 (6.1)	52 (10.5)	0.10
200–400	134 (27.0)	142 (28.7)	148 (29.8)	138 (27.8)	
>400	323 (65.0)	318 (64.2)	313 (63.1)	302 (60.8)	
Alcohol consumption (*n*, %)					
None	121 (24.4)	167 (33.7)	197 (39.7)	231 (46.5)	< 0.001
Ex-drinker	11 (2.2)	22 (4.4)	22 (4.4)	21 (4.2)	
Current drinker	365 (73.4)	306 (61.8)	277 (55.9)	245 (49.3)	
Smoking status (*n*, %)					
None	336 (67.6)	394 (79.6)	415 (83.7)	436 (87.7)	< 0.001
Ex-smoker	66 (13.3)	42 (8.5)	29 (5.9)	25 (5.0)	
Current smoker	95 (19.1)	59 (11.9)	52 (10.5)	36 (7.2)	
Physical activity (MET-min/week) (*n*, %)					
Low	142 (28.6)	144 (29.1)	140 (28.2)	165 (33.2)	< 0.001
Moderate	132 (26.6)	145 (29.3)	165 (33.3)	189 (38.0)	
High	223 (44.9)	206 (41.6)	191 (38.5)	143 (28.8)	
Total caloric intake (kcal/day)	1971.18 ± 626.39	2059.19 ± 665.38	2134.21 ± 730.47	1986.88 ± 752.94	0.94
MetS					
No	417 (83.9)	405 (81.8)	423 (85.3)	438 (88.1)	0.045
Yes	80 (16.1)	90 (18.2)	73 (14.7)	59 (11.9)	
WC (cm)	84.17 ± 9.82	83.02 ± 9.25	82.66 ± 9.32	81.10 ± 9.28	< 0.001
TG (mg/dL)	148.80 ± 164.46	133.20 ± 106.47	131.78 ± 127.30	116.48 ± 82.82	< 0.001
Systolic BP (mmHg)	118.52 ± 15.65	117.15 ± 15.52	116.33 ± 14.14	115.71 ± 15.88	0.004
Diastolic BP (mmHg)	75.62 ± 12.06	73.44 ± 12.26	72.98 ± 11.59	71.83 ± 12.09	< 0.001
FBG (mg/dL)	85.06 ± 16.58	83.91 ± 14.02	84.93 ± 21.82	82.75 ± 10.33	0.20
HDL-C (mg/dL)	55.65 ± 13.90	56.41 ± 13.60	57.06 ± 14.50	58.13 ± 13.49	0.002

BMI, body mass index; BP, blood pressure; FBG, fasting blood glucose; HDL-C, high-density lipoprotein cholesterol; MET, metabolic equivalent unit; MetS, metabolic syndrome; Q, quartile; TG, triglycerides; WC, waist circumference. Values are presented as *n* (%) for categorical variables and means ± SD for continuous variables. Residuals of dietary fiber was used to determine quartile groups. *P* values for trend were calculated with the use of linear regression (BMI), the Jonckheere-Terpstra test (other continuous variables) or the Cochran-Mantel-Haenszel test for categorical variables, where appropriate

### Association between dietary fiber intake and risk of metabolic syndrome including components

Table 2 indicates the results of the multivariable model adjusted for covariables, showing the association between quartiles of fiber intake and the prevalence of MetS with its components. The findings demonstrate that individuals with higher fiber intake had a lower prevalence of increased WC (*P* = 0.005), elevated TG (*P* < 0.001), elevated BP (*P* = 0.007), and the risk of MetS (*P* = 0.025). Furthermore, higher fiber intake was significantly associated with lower ORs of MetS in individuals with elevated WC (OR, 95% CI = 0.60, 0.41–0.88, *P* for trend = 0.009) and elevated TG (OR, 95% CI = 0.69, 0.50–0.96, *P* for trend = 0.012) compared to those in the lowest quartile of fiber intake.

**Table 2:** Multivariable adjusted odds ratio (OR) and 95% confidence intervals (CI) of metabolic syndrome and its components by quartiles of dietary fiber intake

	Q1	Q2	Q3	Q4	*P* for trend
Increased WC					
Prevalence, *n* (%)	168 (8.5)	154 (7.8)	153 (7.7)	126 (6.4)	0.005
Crude OR (95% CI)	1.0 (ref)	0.88 (0.68–1.15)	0.87 (0.67–1.14)	0.67 (0.51–0.88)	0.004
Multivariable OR (95% CI)	1.0 (ref)	0.79 (0.55–1.13)	0.75 (0.52–1.07)	0.60 (0.41–0.88)	0.009
Elevated TG					
Prevalence, *n* (%)	176 (8.9)	159 (8.0)	144 (7.3)	111 (5.6)	< 0.001
Crude OR (95% CI)	1.0 (ref)	0.86 (0.66–1.12)	0.75 (0.57–0.98)	0.53 (0.40–0.69)	< 0.001
Multivariable OR (95% CI)	1.0 (ref)	1.01 (0.75–1.37)	0.82 (0.60–1.11)	0.69 (0.50–0.96)	0.012
Elevated BP					
Prevalence, *n* (%)	153 (7.7)	138 (7.0)	124 (6.3)	118 (5.9)	0.007
Crude OR (95% CI)	1.0 (ref)	0.87 (0.66–1.14)	0.75 (0.57–0.99)	0.70 (0.53–0.93)	0.009
Multivariable OR (95% CI)	1.0 (ref)	1.00 (0.74–1.36)	0.83 (0.61–1.14)	0.96 (0.70–1.32)	0.63
Elevated FBG					
Prevalence, *n* (%)	36 (1.8)	43 (2.2)	32 (1.6)	26 (1.3)	0.10
Crude OR (95% CI)	1.0 (ref)	1.22 (0.77–1.93)	0.88 (0.54–1.45)	0.71 (0.42–1.19)	0.10
Multivariable OR (95% CI)	1.0 (ref)	1.35 (0.83–2.20)	0.92 (0.54–1.55)	0.78 (0.44–1.37)	0.21
Reduced HDL-C					
Prevalence, *n* (%)	96 (4.8)	109 (5.5)	106 (5.3)	115 (5.8)	0.19
Crude OR (95% CI)	1.0 (ref)	1.18 (0.87–1.61)	1.14 (0.83–1.55)	1.26 (0.93–1.71)	0.18
Multivariable OR (95% CI)	1.0 (ref)	1.02 (0.74–1.40)	0.92 (0.67–1.27)	0.96 (0.70–1.33)	0.72
MetS					
Prevalence, *n* (%)	80 (4.0)	90 (4.5)	73 (3.7)	59 (3.0)	0.025
Crude OR (95% CI)	1.0 (ref)	1.16 (0.83–1.61)	0.90 (0.64–1.27)	0.70 (0.49–1.01)	0.022
Multivariable OR (95% CI)	1.0 (ref)	1.32 (0.90–1.92)	0.90 (0.61–1.33)	0.81 (0.53–1.24)	0.14

BP, blood pressure; FBG, fasting blood glucose; HDL-C, high-density lipoprotein cholesterol; MetS, metabolic syndrome; Q, quartile; TG, triglycerides; WC, waist circumference. Residuals of dietary fiber was used to determine quartile groups. *P* values for trends were calculated with the use of the Cochran-Amitage test (for the prevalence estimates) or logistic regression (by treating the order of the quartile group as a continuous variable). Multivariable model was adjusted for age, sex, BMI, education level, occupation, income, alcohol consumption, smoking status, and physical activity

### Association between *APOA5* genetic variants and metabolic syndrome risk

Table 3 presents the association between *APOA5* genetic variants (rs2266788, rs662799, and rs651821) and MetS in the multivariable model. The minor allele frequencies of the three SNPs were common (MAF > 5%); rs2266788 (0.13), rs662799 (0.16), and rs651821 (0.18). These SNPs in the non-MetS groups were in HWE. In the multivariable model adjusted for covariates, those who were homozygous for rs662799 (OR, 95% CI = 2.59, 1.66–4.04, *P* < 0.001, G/G vs. A/A) and rs651821 (OR, 95% CI = 2.64, 1.69–4.13, *P* < 0.001, C/C vs. T/T) had an increased risk of MetS. When comparing the genetic models of *APOA5* variants, the dominant models of the three variants of the *APOA5* gene showed a significant association with MetS: carrying a G allele of rs2266788 (OR, 95% CI = 1.37, 1.03–1.81, *P* = 0.029, A/G + G/G vs. A/A), carrying a G allele of rs662799 (OR, 95% CI = 1.74, 1.31–2.31, *P* < 0.001, A/G + GG vs. A/A), and carrying a C allele of rs651821 (OR, 95% CI = 1.76, 1.32–2.33, *P* < 0.001, T/C + C/C vs. T/T). In the recessive model, those who carried a G allele of rs662799 (OR, 95% CI = 2.09, 1.37–3.17, *P* < 0.001, G/G vs. A/A + A/G) and a C allele of rs651821 (OR, 95% CI = 2.12, 1.39–3.22, *P* < 0.001, C/C vs. T/T + T/C) had an increased risk of MetS.

**Table 3:** Association between *APOA5* genetic variants and metabolic syndrome risk

APOA5	Non-MetS/MetS	Crude OR (95% CI)	*P*-value	Multivariable OR (95% CI)	*P*-value
rs2266788					
A/A	1051/171	1.0 (ref)		1.0 (ref)	
A/G	546/114	1.28 (0.99–1.66)	0.06	1.37 (1.02–1.83)	0.038
G/G	81/17	1.29 (0.75–2.23)	0.36	1.37 (0.75–2.51)	0.30
Dominant					
A/A	1051/171	1.0 (ref)		1.0 (ref)	
A/G + G/G	627/131	1.28 (1.00-1.65)	0.048	1.37 (1.03–1.81)	0.029
Recessive					
A/A + A/G	1597/285	1.0 (ref)		1.0 (ref)	
G/G	81/17	1.18 (0.69–2.01)	0.55	1.23 (0.68–2.21)	0.50
rs662799					
A/A	877/123	1.0 (ref)		1.0 (ref)	
A/G	663/136	1.46 (1.12–1.90)	0.005	1.57 (1.16–2.12)	0.003
G/G	140/42	2.14 (1.44–3.17)	< 0.001	2.59 (1.66–4.04)	< 0.001
Dominant					
A/A	877/123	1.0 (ref)		1.0 (ref)	
A/G + G/G	803/178	1.58 (1.23–2.03)	< 0.001	1.74 (1.31–2.31)	< 0.001
Recessive					
A/A + A/G	1540/259	1.0 (ref)		1.0 (ref)	
G/G	140/42	1.78 (1.23–2.58)	0.002	2.09 (1.37–3.17)	< 0.001
rs651821					
T/T	863/121	1.0 (ref)		1.0 (ref)	
T/C	665/137	1.47 (1.13–1.91)	0.004	1.58 (1.17–2.13)	0.003
C/C	139/42	2.16 (1.45–3.20)	< 0.001	2.64 (1.69–4.13)	< 0.001
Dominant					
T/T	863/121	1.0 (ref)		1.0 (ref)	
T/C + C/C	804/179	1.59 (1.24–2.04)	< 0.001	1.76 (1.32–2.33)	< 0.001
Recessive					
T/T + T/C	1528/258	1.0 (ref)		1.0 (ref)	
C/C	139/42	1.79 (1.24–2.59)	0.002	2.12 (1.39–3.22)	< 0.001

MetS, metabolic syndrome; CI, 95% confidence intervals; OR, odds ratios. *P*-values were calculated using the chi-square test. Multivariable model was adjusted for age, sex, BMI, education level, occupation, income, alcohol consumption, smoking status, and physical activity

### Interaction between dietary fiber intake and *APOA5* variants regarding metabolic syndrome risk

Table 4 demonstrates the interaction between dietary fiber consumption and MetS risk according to the genetic models of *APOA5* variants. To investigate the gene-diet interaction, dietary fiber intake was divided into low and high groups based on the median levels in the non-MetS groups. When comparing the genetic models of each variant, low fiber intake while carrying a G allele of rs662799 showed an increased risk of MetS compared to that of individuals who were AA carriers in the dominant model, indicating a stronger interaction (OR, 95% CI = 2.34, 1.59–3.44, *P* for interaction = 0.024, A/G + G/G carriers with low fiber intake vs. A/A carriers with low fiber intake). Similarly, the significant association between dietary fiber intake and the risk of MetS was stronger among carriers of the rs651821 C allele than the T/T homozygous variant in the dominant model (OR, 95% CI = 2.35, 1.59–3.46, *P* for interaction = 0.027, T/C + C/C carriers with low fiber intake vs. T/T carriers with low fiber intake).

**Table 4:** Interaction between dietary fiber intake and *APOA5* genetic variants regarding metabolic syndrome risk

APOA5	Non-MetS/MetS		Crude OR (95% CI)		*P* for interaction	Multivariable OR (95% CI)		*P* for interaction
SNPs	Low	High	Low	High		Low	High	
rs2266788								
Dominant								
A/A	527/96	524/75	1.0 (ref)	0.79 (0.57–1.09)	0.63	1.0 (ref)	0.78 (0.54–1.13)	0.69
A/G + G/G	312/77	315/54	1.36 (0.97–1.89)	0.94 (0.66–1.35)		1.43 (0.98–2.09)	1.00 (0.66–1.51)	
Recessive								
A/A + A/G	792/163	805/122	1.0 (ref)	0.74 (0.57–0.95)	0.62	1.0 (ref)	0.74 (0.55–0.99)	0.78
G/G	47/10	34/7	1.03 (0.51–2.09)	1.00 (0.44–2.30)		1.13 (0.52–2.46)	0.98 (0.40–2.42)	
rs662799								
Dominant								
A/A	447/63	430/60	1.0 (ref)	0.99 (0.68–1.44)	0.05	1.0 (ref)	1.07 (0.70–1.63)	0.024
A/G + G/G	393/109	410/69	1.97 (1.40–2.76)	1.19 (0.83–1.72)		2.34 (1.59–3.44)	1.30 (0.86–1.98)	
Recessive								
A/A + A/G	770/149	770/110	1.0 (ref)	0.74 (0.57–0.96)	0.77	1.0 (ref)	0.73 (0.54–0.99)	0.79
G/G	70/23	70/19	1.70 (1.03–2.81)	1.40 (0.82–2.40)		1.99 (1.12–3.53)	1.62 (0.89–2.96)	
rs651821								
Dominant								
T/T	441/62	422/59	1.0 (ref)	0.99 (0.68–1.46)	0.06	1.0 (ref)	1.07 (0.70–1.63)	0.027
T/C + C/C	392/109	412/70	1.98 (1.41–2.78)	1.21 (0.84–1.75)		2.35 (1.59–3.46)	1.32 (0.87–2.01)	
Recessive								
T/T + T/C	764/148	764/110	1.0 (ref)	0.74 (0.57–0.97)	0.81	1.0 (ref)	0.73 (0.54-1.00)	0.85
C/C	69/23	70/19	1.72 (1.04–2.85)	1.40 (0.82–2.40)		2.05 (1.15–3.66)	1.63 (0.89–2.98)	

MetS, metabolic syndrome; CI, 95% confidence intervals; OR, odds ratios. Dietary fiber intake was categorized into low and high groups based on the median level. *P*-values were calculated using the chi-square test. Multivariable model was adjusted for age, sex, BMI, education level, occupation, income, alcohol consumption, smoking status, and physical activity

## Discussion

The present study evaluated the association between dietary fiber consumption and the prevalence of MetS according to the genetic variants of the *APOA5* gene among Korean adults. The findings indicated that higher dietary fiber intake showed a lower prevalence of MetS risk. Among the components of MetS, increased WC and elevated TG were associated with dietary fiber intake. Moreover, *APOA5* rs662799 and rs651821 variants were found to modify the associations between dietary fiber intake and the risk of MetS, depending on genotype.

Previous epidemiological studies have shown conflicting results. This might be because of the different study populations (races), ages (adolescents or elderly adults), and dietary assessment methods, as Chen et al. mentioned in a meta-analysis on dietary fiber and MetS [9]. Furthermore, genetic heterogeneity or the gut microbiome in the putative processes underlying fiber and MetS may influence how dietary fiber affects the disease [33]. Recent evidence from a wide range of mechanistic studies relevant to MetS reveals an inverse association between dietary fiber intake and MetS, which is consistent with the findings of the present study [9]. MetS is caused by the co-occurrence of metabolic abnormalities, including obesity, insulin resistance, dyslipidemia, and hypertension. Plausible mechanisms of the dietary fiber effect on each component of MetS have been suggested. In the case of central obesity, appetite regulation and energy homeostasis may be linked to dietary fiber intake [34–36]. The insulin resistance and glucose tolerance associated with the modulation of glycemia and insulinemia-lowering properties could also be attributed to fiber consumption [37, 38]. The cholesterol-lowering effect of dietary fiber may result from the increase in the total excretion of fecal bile acids and the decrease in the glycemic response [39, 40]. Regarding the effect of dietary fiber on hypertension, several observational studies have shown an inverse association between them [41, 42], but the biological mechanism underlying this relationship remains unclear. In this study, we observed that high consumption of dietary fiber reduced the risk of increased WC and elevated TG levels. A meta-analysis of randomized controlled trials reported that dietary viscous fiber significantly affects decreased body weight and BMI, particularly WC [43]. Accumulating evidence suggests that increased intake of dietary fiber contributes to the regulation of energy metabolism by diluting excess energy, decreasing the absorption rate, and stimulating appetite suppression [44, 45]. On the other hand, evidence in the literature suggests that fiber intake may contribute to a hypocholesterolemic effect while not affecting hypotriglyceridemia [46, 47]. Nevertheless, the potential mechanisms suggest that metabolic responses affect the bioavailability of fiber based on the physicochemical characteristics of fiber in the lipid profile [48, 49]. Recently, several studies have suggested the benefit of dietary fiber consumption on gut bacteria, as high intake of fiber could modify gut microbiota populations, leading to a reduction in the risk of MetS [50, 51]. However, there are still insufficient studies to support and verify that fiber extensively mediates protective effects on the risk of MetS.

Despite the low plasma concentration of APOA5, its major function is to regulate plasma TG levels by enhancing TG-rich lipoprotein metabolism, inhibiting VLDL particle production, and accelerating the hepatic uptake of lipoprotein remnants [12]. The extracellular role of APOA5 is to facilitate plasma TG hydrolysis by stimulating lipoprotein lipase activity [52]. The plasma TG levels were significantly increased in transgenic APOA5 knockout mice, whereas the level of TG was decreased in mice overexpressing the *APOA5* gene [11]. The intracellular role of the *APOA5* gene is associated with cytoplasmic lipid droplets and has been proven to modulate TG storage in adipocytes [53]. Based on these physiological functions, recent study results have identified the link between the *APOA5* gene and metabolic disorders, including obesity and MetS [54]. In this study, three common genetic variants in the *APOA5* gene (rs2266788, rs662799, and rs651821) were associated with the risk of MetS. Several studies were conducted to explore the association between rs2266788 (3’-UTR variant) and TG levels conferring MetS risk across ethnic differences [54–56]. According to a longitudinal prospective cohort study in a Korean population, rs2266788 showed a significant correlation with MetS susceptibility [57]. Accumulating evidence has also determined that rs662799 genetic variants located upstream of the *APOA5* promoter are linked to hypertriglyceridemia and increase the risk of MetS with its individual components [18, 20, 58, 59]. Additionally, the polymorphisms of rs662799 and rs651821 (5’-UTR variant) indicated significant associations with familial combined hyperlipidemia and lipid traits [60]. Zhou et al. reported that polygenetic variants at the 11q23 locus, including *APOA5* rs662799, are linked to an increased 3GO risk of a condition called co-occurring hypertension, hyperglycemia, and dyslipidemia, which is composed of MetS [61]. The 3GO risk of the haplotype’s minor alleles was higher for the subjects who consumed less dietary fiber than it was for the major alleles, demonstrating the influence of interactions between the 11q23 haplotype and environmental factors [61]. The SNPs in the *APOA1/C3/A4/A5* cluster showed interactions with environmental factors for the risk of MetS and its components [62]. Park et al. identified that carrying the minor alleles of rs2266788 and rs662799 increased the risk of TG levels showing interactions with specific nutrients (low fat, high carbohydrate, and low calcium), alcohol, and smoking status in men [24]. In terms of modifiable lifestyle factors, the incidence of MetS and its lipid-influencing components in the minor allele of the rs662799 variant had strong interactions with red and processed meat intake, a low-calorie diet with a Mediterranean pattern, physical activity, alcohol consumption, and smoking compared to the involvement of the major allele of this genetic variant [25, 63, 64]. In a recent study, Lim et al. demonstrated that altered gut microbes mediated by the *APOA5* rs651821 variant were associated with the risk of MetS [65]. In this study, the risk of MetS was shown to be significantly increased in both the dominant and recessive genetic models of *APOA5* variants. However, when comparing the interaction between each genetic model of *APOA5* variants and dietary fiber intake, the impact on MetS susceptibility was different. In the dominant model of *APOA5* variants, low dietary fiber intake was associated with an increased risk of MetS with a G allele of rs662799 and a C allele of rs651821 compared to high fiber intake. This finding indicates that the effects of dietary fiber on the risk of MetS were different for *APOA5* rs662799 and rs651821 variants.

The possible mechanisms of how fiber regulates the risk of MetS by *APOA5* rs662799 and rs651821 could be explained by the benefits of fiber on hyperlipidemia and hyperglycemia according to genetic variants. According to the evidence on the characteristics of fiber, such as a lower calorie content, bulking effect, and physicochemical properties (e.g., solubility and viscosity), fiber consumption delays gastric emptying in the stomach and reduces the absorption of lipids and carbohydrates in the intestine, resulting in lower TG levels [66, 67]. Given that APOA5 plays a role in the metabolism of TG-rich particles, high consumption of fiber may result in less activation of lipoprotein lipase in TG catabolism. High consumption of fiber may also be linked to reducing the chylomicron and hepatic synthesis of VLDL and its remnants [68]. Cicero et al. indicated that β-glucans, one of the bioactive compounds in fiber, contribute to the formation of a gelatinous layer in the intestinal lumen, leading to inhibition of lipid absorption and then lowering the effects of TG and cholesterol [69]. In addition, based on the fermentability of fiber, β-glucans impact bacterial metabolism and increase the diversity of gut microbiota linked to obesity and metabolic responses [70, 71]. The current study suggests the role of *APOA5* genetic variants in determining the responses of MetS and obesity to dietary fiber related to plasma TG levels. However, our understanding of the molecular mechanisms of *APOA5* gene expression that are responsible for the risk of MetS, including the antihyperlipidemic and antihyperglycemic benefits of fiber, is limited.

Several limitations should be considered when interpreting the results. Despite the inverse associations between dietary fiber consumption and the risk of MetS, the cross-sectional design of this study confers an inability to determine a temporal and causal inference. Moreover, owing to providing information on the prevalence of MetS and fiber intake in a population, it is difficult to assess the incidence or changes in MetS and long-term fiber intake over time. Dietary fiber consumption was collected using the SQFFQ based on habitual diet, but use of this tool includes potential recall bias, and the results cannot entirely reflect the participant’s usual intake. The energy adjusted for fiber by the residual method was used to assess and minimize measurement errors [32]. Although the current study observed interactions between dietary fiber intake and *APOA5* variants for the risk of MetS, further study is needed to examine a sufficiently large population to improve statistical power and to carefully select relevant genetic markers to ensure the validity and reliability of the findings. Additionally, there might be potential and unmeasured confounding factors, which may affect the association between dietary fiber intake and MetS according to *APOA5* genetic variants, although this study considered relevant confounders, including age, sex, sociodemographic, and lifestyle factors. Subsequent investigations utilizing large-scale studies involving representative population-based cohorts are necessary to substantiate the causal association across diverse ethnic backgrounds and geographic regions. A sufficiently substantial sample size has the capacity to detect meaningful genetic effects and gene-diet interactions with the requisite statistical power. To elucidate the interplay of fiber intake and *APOA5* variants in relation to MetS risk, the exploration of additional pertinent genetic variants and the examination of the enduring effects of these interactions are imperative. Furthermore, the implementation of functional studies elucidating molecular mechanisms may significantly contribute to augmenting the generalizability of the identified interactions involving dietary fiber intake and MetS altering *APOA5* variants.

Based on the findings of gene-diet interactions in this study, we found that metabolic processes (e.g., TG metabolism), absorption, and responsiveness, may exhibit variations contingent upon the individual’s *APOA5* genetic variant. Consequently, nutritional strategies can be formulated based on these genetic considerations to reflect the individual’s specific requirements and sensitivities to dietary fiber. The enhancement of individual dietary practices is achievable through regulation of the quantity and proportion of dietary fiber influenced by the *APOA5* genetic variant, which facilitates the provision of optimized nutritional guidelines aimed at promoting optimal nutrient metabolism, absorption, and overall lifestyle.

In conclusion, the response of MetS and its components to dietary fiber may be altered by individual genetic variants. Given that there were interactions between dietary fiber intake and *APOA5* variants (rs662799 and rs651821) for the risk of MetS, we propose that low consumption of fiber may impact TG metabolism linked to MetS risk by altering the *APOA5* gene. Furthermore, the understanding of how dietary fiber interacts with the risk of MetS and *APOA5* genetic variants could provide more evidence to clarify the related molecular mechanisms and provide an effective strategy for preventing MetS.

### Electronic supplementary material

Below is the link to the electronic supplementary material.

Supplementary Material 1

## Data Availability

Due to ethical restrictions approved by the ethics committee of our institution, the data used in this study can be made available for research proposals by a request to Proof’s Publications Committee.

## List of abbreviations

APOA5: Apolipoprotein A5
BMI: Body mass index
BP: Peripheral blood pressure
CI: Confidence intervals
FBG: Fasting blood glucose
GWAS: Genome-wide association studies
HDL-C: High-density lipoprotein cholesterol
HWE: Hardy-Weinberg equilibrium
KDCC: The Korean Medicine Daejeon Citizen Cohort
LDL: Low-density lipoprotein
MAF: Minor allele frequency
MET: Metabolic equivalent task
MetS: Metabolic syndrome
NCEP-ATP III: The National Cholesterol Education Program-Adult Treatment Panel III
OR: Odds ratios
SNP: Single nucleotide polymorphism
SQFFQ: Semiquantitative food frequency questionnaire
TG: Triglycerides
VLDL: Very low-density lipoprotein
WC: Waist circumference
