# Supplementary material for: Consumption of dietary fiber and APOA5 genetic variants in metabolic syndrome: baseline data from the Korean Medicine Daejeon Citizen Cohort Study
Source: Nutr Metab (Lond). 2024 Apr 5;21:19. doi: 10.1186/s12986-024-00793-0 (PMC10998362; doi:10.1186/s12986-024-00793-0)
Supplement: Supplementary file 1 — Supplementary Material 1 [file 12986_2024_793_MOESM1_ESM.docx]

**Supplementary table S1**. Description of SNPs in APOA5 gene ^1^

| **rs number** | **Chromosome**  **location** | **Allele**  **(Major/Minor)** | **Functional consequence** | **MAF ^2^** | **HWE**  ***P*-value** | **Ref** |
| --- | --- | --- | --- | --- | --- | --- |
| rs2266788 | 11q23.3 | A/G | 3’- UTR variant | 0.13 | 0.35 | [16,17,18] |
| rs662799 | 11q23.3 | A/G | Upstream transcript variant | 0.16 | 0.35 | [18,19] |
| rs651821 | 11q23.3 | T/C | 5’- UTR variant | 0.18 | 0.50 | [17,19] |

MAF, Minor allele frequency; HWE, Hardy-Weinberg equilibrium. ^1^ Data on the NCBI dbSNP database (<http://www.ncbi.nlm.nih.gov/projects/SNP>) and the GWAS Catalog (https://www.ebi.ac.uk/gwas/). ^2^ Minor allele frequency in the non-metabolic syndrome groups of this study.
